# Habitual exercise in youth: A ʻbrainyʼ idea

**DOI:** 10.1113/EP091534

**Published:** 2023-11-02

**Authors:** Jill N. Barnes, Tracy Baynard, Patrice Brassard

**Affiliations:** ^1^ Bruno Balke Biodynamics Laboratory University of Wisconsin‐Madison Madison Wisconsin USA; ^2^ Integrative Human Physiology Laboratory University of Massachusetts Boston Boston Massachusetts USA; ^3^ Department of Kinesiology, Faculty of Medicine Université Laval Québec Canada; ^4^ Research Center of the Institut Universitaire de Cardiologie et de Pneumologie de Québec Québec Canada

**Keywords:** cerebral blood flow, cerebrovascular reactivity, exercise, hypercapnia, maturation, paediatric

1

The impact of exercise training on cerebrovascular health is a hot topic, with an increasing focus on preventing and delaying neurodegenerative diseases. Yet, there is a paucity of information regarding the effect of habitual exercise on cerebrovascular function, particularly in children and adolescents. This age range is often under‐represented in research but can offer important insight concerning the trajectory of health. It is also unclear what the consequences of a lack of habitual exercise are for cerebrovascular health in youth.

To address this gap in the literature, in an article in this issue of *Experimental Physiology*, Talbot et al. investigated both cerebral blood flow (CBF) and cerebrovascular reactivity to carbon dioxide in youth, while accounting for different stages of maturation (using a somatic measure of maturity – predicted age at peak height velocity or PHV) (Talbot, Perkins, Tallon et al., 2023). This study required a considerable number of participants to account for sex, maturation and training status. The authors reported novel findings that endurance‐trained youth had higher global CBF at rest (using internal carotid artery (ICA) + vertebral artery blood flow assessments) in comparison to untrained counterparts. In the ʻmatureʼ adolescents, cardiorespiratory fitness was associated with global CBF. *Post hoc* analysis revealed that untrained post‐PHV (or ʻmatureʼ) males demonstrated lower global CBF compared with trained post‐PHV males, with no effect of training found in post‐PHV females or younger (pre‐PHV) groups. Untrained youth were defined as ʻnot taking part in regular exerciseʼ or not meeting established guidelines for physical activity. Although difficult to directly compare, the global CBF values in untrained post‐PHV males were similar to middle‐aged males in a recent study that quantified global CBF in a similar manner (Tomoto et al., 2023).

A group of these authors, who commonly study maturation, have recently shown that step count was lower and sedentary time was higher with maturation in children (Tallon et al., 2021). Interestingly, when children and adolescents were grouped into tertiles by sedentary time, higher sedentary time corresponded to lower ICA shear rate. Coupled with the most recent findings from Talbot, Perkins, Tallon et al. (2023), this suggests that exercise training in adolescents may be necessary to counteract the negative consequences of higher sedentary time on cerebral haemodynamics.

While there is variability in CBF measures between maturation stages in males and females, and now with exercise training status, this emphasizes the need for understanding longitudinal CBF changes in youth. It is known that global CBF decreases between childhood and adolescence (Wang et al., 2003), but how normal physiological declines in CBF that occur with maturation differ from pathological declines in CBF is unclear. Twin studies in adolescents and young adults suggest only moderate heritability in CBF (Dang et al., 2023). Accordingly, environmental or behavioural factors, particularly during this stage of brain development, may be a critical component of cerebrovascular health. Therefore, elevated sedentary time coupled with a lack of habitual exercise during maturation may have a significant impact on CBF, brain development and brain volume later in adulthood.

Additional beneficial adaptions of habitual exercise may only be ʻrevealedʼ when the system is stressed, rather than by investigating CBF at rest (Brugniaux et al., 2014). The study by Talbot et al. investigated how maturation, sex and training status were related to the cerebrovascular response to steady state elevations in carbon dioxide (CO_2_) (Talbot, Perkins, Tallon et al., 2023). While there were no significant training effects on cerebrovascular reactivity to hypercapnia, the authors reported that pre‐PHV‐trained children had a faster cerebrovascular response compared with the untrained group. In a separate study by Talbot et al., they reported that pre‐PHV‐trained children had a smaller increase in cerebral pulsatility of the posterior cerebral artery (PCA) during a visual stimulus, compared with untrained pre‐PHV children (Talbot, Perkins, Dawkins et al., 2023). The interpretation is that trained children had better dampening function in the PCA, compared with untrained children. Thus, both findings from examining the cerebrovascular kinetics during a ʻstressʼ revealed exercise training adaptations in the pre‐PHV cohort, prior to maturation.

The faster ICA mean response time to hypercapnia in pre‐PHV children (Talbot, Perkins, Tallon et al., 2023) is an interesting finding and brings to light questions for the field. For example, what is the physiological or clinical significance of a faster response to hypercapnia in children prior to reaching PHV or maturation? Previous studies in young adults investigated middle cerebral artery blood velocity (MCAv) kinetic responses during the onset of exercise and reported no effect of cardiorespiratory fitness on the MCAv kinetics (Weston et al., 2022). Other studies in older or clinical populations that investigated MCAv kinetics reported a blunted response to acute exercise in older adults (Ward et al., 2018) and after ischaemic stroke (Kaufman et al., 2019). Collectively, these studies investigating the MCAv kinetic response to exercise suggest that a slower response may indicate an ʻimpairmentʼ. It is less clear whether a faster ICA response time to hypercapnia represents a beneficial adaptation.

An additional question regarding the cerebrovascular response to CO_2_ is the utilization of a single trial to characterize the kinetic response. In the oxygen uptake kinetics literature, several trials will usually be performed by participants at the same exercise intensity (e.g. 80% of ventilatory threshold) to improve the signal‐to‐noise ratio and the subsequent modelling of the time constant (Lamarra et al., 1987). Given the variability of the cerebrovascular responses to CO_2_, and that the ventilatory response to hypercapnia would influence the ICA results, would it have been necessary to average several trials to improve confidence in the kinetic modelling? Nonetheless, these novel findings, coupled with previous work from Tallon et al. represent a major step forward in the study of cerebrovascular responses to CO_2_ in youth and adolescents (Tallon et al., 2022).

In summary, this comprehensive study provided detailed cerebrovascular measurements in children and adolescents and investigated the potential role of habitual endurance exercise in youth and across maturation stages. These findings provide the groundwork for future studies to investigate the influence of habitual exercise on other cerebrovascular variables. Beyond future research studies, these findings highlight the importance of youth exercise and/or sport participation on cerebrovascular health.

## AUTHOR CONTRIBUTIONS

All authors have approved the final version of the manuscript and agree to be accountable for all aspects of the work in ensuring that questions related to the accuracy or integrity of any part of the work are appropriately investigated and resolved. All persons designated as authors qualify for authorship, and all those who qualify for authorship are listed.

## CONFLICT OF INTEREST

No competing interests are declared.

## FUNDING INFORMATION

No funding was received for this work.
